# Assessment of non-carious root surface defects in areas of gingival recession: A descriptive study

**DOI:** 10.4317/jced.52831

**Published:** 2016-10-01

**Authors:** Vanaja-Krishna Naik, Caroline-Annette Jacob, Deepavalli-Arumuga Nainar

**Affiliations:** 1 MDS, MFDSRCPS [Glasg, UK], Department of Periodontics, SRM Dental College, Bharathi Salai, Ramapuram Chennai; 2 MDS, Department of Periodontics, Saveetha Dental College, Thiruverkadu, Chennai; 3 MDS, Department of Periodontics, Raga’s Dental College, Uthandi, Chennai

## Abstract

**Background:**

The purpose of this descriptive study was to observe the distribution of four different classes of non-carious cervical root surface discrepancies in teeth with gingival recession. Additionally to explore the different treatment modalities in the literature for each of these defects.

**Material and Methods:**

A total of 150 subjects with at least one labial gingival recession were included in the study. 1400 teeth were evaluated using 2.5 X magnification loupes and UNC -15 probe for the presence of the cemento-enamel junction and step like defects according to Pini-Prato’s classification: A-, identifiable CEJ without defect; A+, identifiable CEJ with defect; B-, unidentifiable CEJ without defect, B+, unidentifiable CEJ with defect. Further a comprehensive electronic and hand search of pubmed indexed journals was performed to identify appropriate treatment modalities for these defects and their predictability following restorative/surgical or combination of both.

**Results:**

A total of 1400 teeth with exposed root surfaces were examined (793 Maxillary; 607 mandibular). 499 teeth were A-, 405 were A+, 322 were B+ and 174 were B-. The distribution of these defects in different teeth was: 36% premolars, 32% molars, 21% incisors and 11% canines, collectively 68% in the aesthetic zone.

**Conclusions:**

Majority of these lesions are in the maxillary aesthetic zone. Hence the presence of the CEJ and the defect must be taken into account while managing these defects surgically.

** Key words:**Cervical abrasion, gingival recession, magnification loupes, root coverage, step defects.

## Introduction

Gingival recession is a well-documented consequence of periodontal disease (1). Additionally, several factors are responsible for gingival recession such as toothbrush trauma, (2) malposition of teeth in the arch, (3) thin tissue overlying the root surface (4) and muscle pull (5). Sometimes, even iatrogenic factors such as periodontal therapy (6) or movement of the teeth during orthodontic treatment may lead to gingival recession (4). The classic studies by Löe H *et al.* (7) on Sri Lankan tea labourers and Norwegian academicians suggested that dentally-motivated academicians had gingival recession on the labial surface in early adult life. By the age of 50 years, over 90% of the academic population presented with gingival recession. An interesting finding was, 25% of these were on the buccal surfaces and only 4% in interproximal sites. However, in the Sri Lankan population, although the recession started in early adult life, there was a greater evidence of recession occurring on all tooth surfaces with 70% of labial surfaces and 40% of interproximal surfaces by the age of 50. This goes on to suggest that populations with poor levels of oral hygiene exhibit higher interproximal destruction as well as buccal recession as a consequence of periodontal disease causing loss of attachment. On the contrary, the pattern of recession in academicians may be easily attributed to toothbrush trauma.

The primary concerns of patients with these lesions could be either root dentin sensitivity or aesthetic issues or both which demands intervention with surgical, restorative or combined approach. However the surgical management becomes challenging when these lesions present with an unidentifiable cemento-enamel junction (CEJ) and a root surface discrepancy (8).

Several classifications of gingival recession have been proposed and the most widely used classification is Miller’s (9). This classification is based on the extent of damage to the periodontal apparatus and designed to predict the final outcome of root coverage procedures. However it does not take into account the gingival biotype and the CEJ, which is a fixed reference point. Besides this classification cannot be applied to the areas of palatal recession due to the absence of muco-gingival junction (10).

Since the CEJ is frequently used fixed reference point to evaluate root coverage/ percentage of root coverage, Zuchelli G *et al.* (11) 2006 suggested a method to predetermine the level of root coverage in anteriors and premolars. Subsequently Pini Prato *et al.* (8) in 2010 proposed a classification which describes the dental surface defects that are of significance in diagnosing areas of gingival recessions. Based on this context, we aimed to assess the prevalence and distribution of root surface defects in South Indian population. In addition, we aimed to suggest different treatment approaches for each of these surface defects based on the existing literature evidence.

## Material and Methods

This descriptive study assessed the distribution of the root surface defects as proposed by Pini Prato *et al.* (8) 2010 ( Table 1). The study protocol was approved by the Institutional Ethical Committee [File no: SRMU/M&HS/SRMDC/2010/ OS-STAFF/101]. Participants were explained about the nature of the study and informed written consent was obtained. A total 150 patients (88 males and 62 females) presenting with at least one labial/buccal gingival recession were recruited. This study was conducted in the Department of Periodontics from August 2010 to March 2012.

**Table 1:**
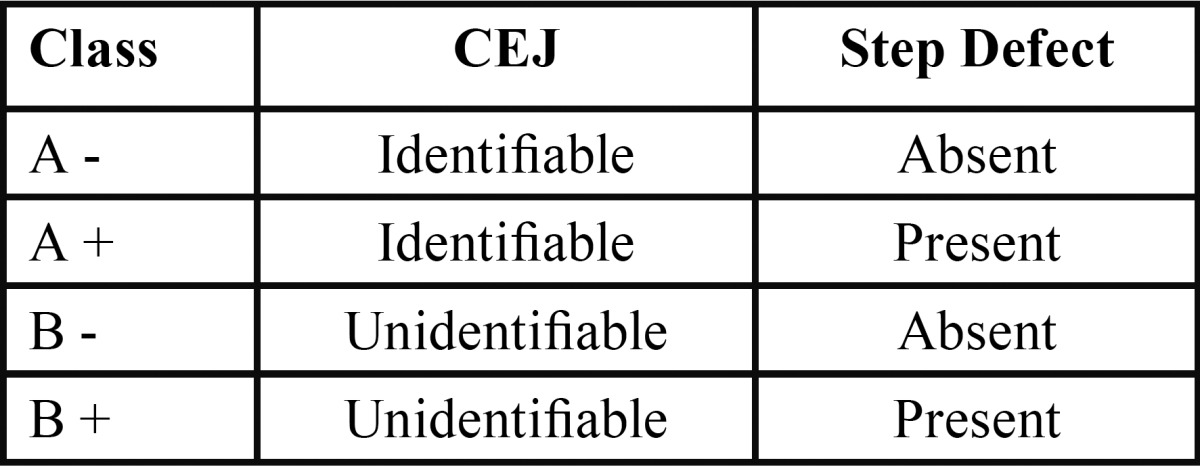
Classification of root surface defects by Pini- Prato 2010.

The individuals selected for the study based on the following eligibility criteria:

Inclusion criteria:

1. Individuals with at least 20 natural teeth

2. Presence of at least one labial recession

3. Non carious teeth

4. Less than 20% plaque and bleeding scores

Exclusion criteria:

1. Medically non compromising systemic health

2. History of periodontal surgery of the affected sites in the past 24 months

3. Smokers

4. Presence of cervical restoration in the area of interest

All new patients were screened for the presence of at least one labial gingival recession, 2 days in a week for 18 months. The individuals selected to be part of the study were based on the eligibility criteria.

Sample size calculation: The sample size was calculated based on the prevalence of the disease taken from the hospital records and methodology assessment with the power of 90 and error 5%. Samples were collected through non probability sampling method.

The calibration of the examiner was done prior to the start of the study. Firstly examiner absorbed, understood and learnt the classification system by Pini-Prato 2010 and then practiced on the same group of 25 patients at two different time points (consecutive 2 days). This initial representative sample was selected by the other two clinicians (CAJ and DAN). Furthermore the intra-examiner reproducibility was calculated using kappa statistics with the value of 0.80 indicating substantial agreement.

Search strategy: The searches were limited to titles, abstracts and papers in English. The search essentially covered scientific databases including PubMed, Medline and Google scholar for articles. Additionally, manual hand searches were also carried out for journals. The journals included in this study were Journal of Clinical Periodontology, Journal of Periodontology, International Journal of Periodontics and Restorative Dentistry and Journal of Indian Society of Periodontology. The articles included were systematic reviews, randomized controlled clinical trials, controlled clinical trials and case series.

A total of 1400 teeth with areas of gingival recessions in both the maxillary and mandibular arches were evaluated for the root surface defects after a thorough oral prophylaxis. The instruments used were UNC-15 periodontal probe, mouth mirror, dental explorer and 2.5 X magnification loupes. The clinical parameters assessed were the identification of the CEJ and a root surface defect caused by cervical wear, by a single calibrated examiner.

## Results

**Figure 1:**
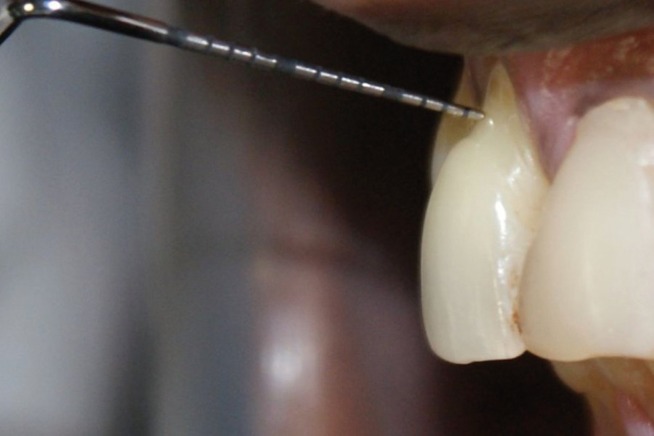
A positive.

**Figure 2:**
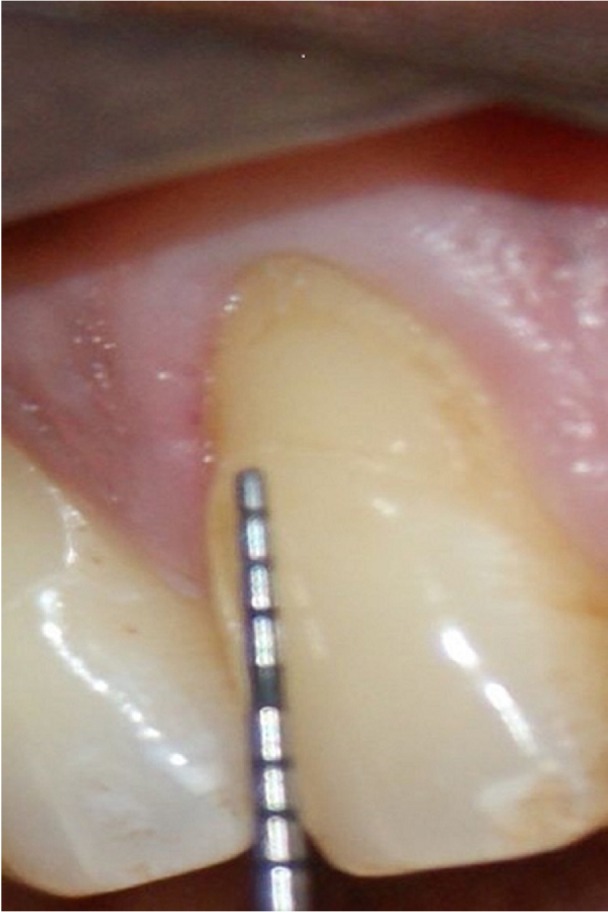
A negative.

**Figure 3:**
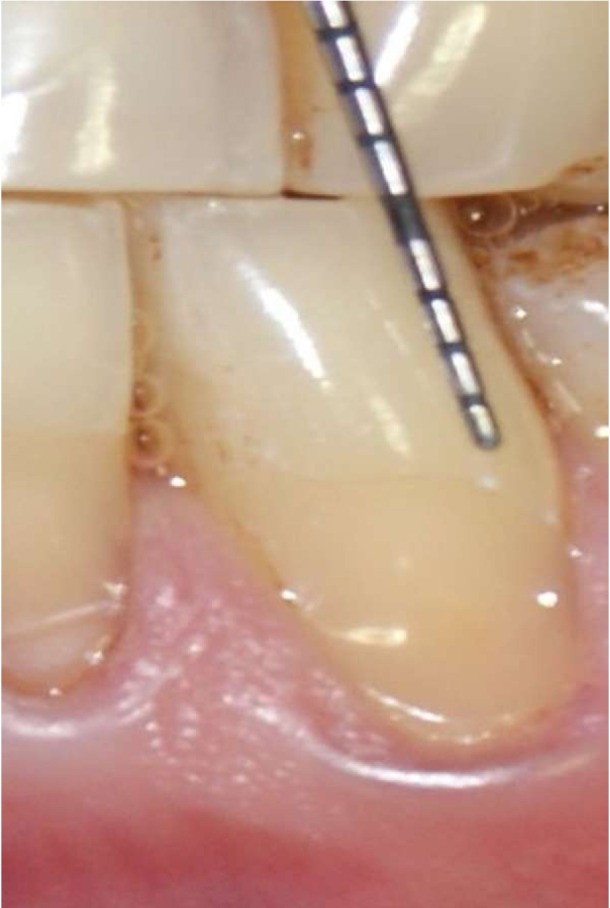
B positive.

**Figure 4:**
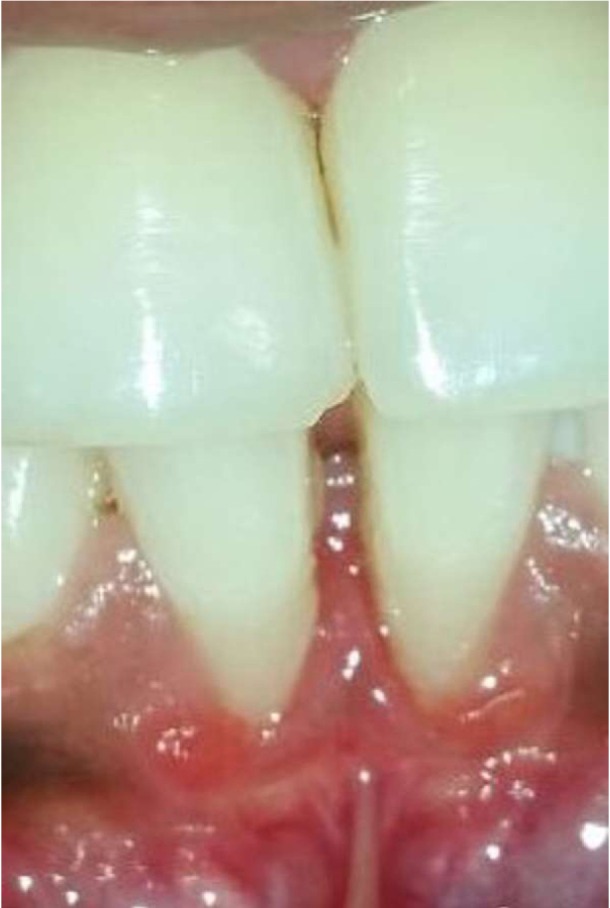
B negative.

A total of 1400 exposed root surfaces were examined to detect the presence of CEJ and the associated root surface defect. The maxillary and mandibular arch showed 793 and 607 teeth with areas of recession respectively (Figs. 1-4). Out of these teeth, 36% were premolars; 32% molars; 21% incisors and 11% canines ( Table 2). A total of 68% of these areas were in the aesthetic zone.

**Table 2:**
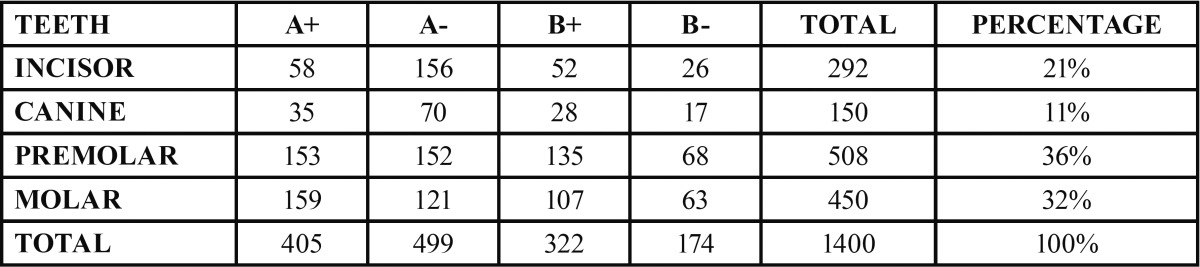
Percentage of root surface defects in different teeth type.

The class A – defects were observed in 499 teeth (284 maxillary and 215 mandibular teeth) which constituted 36% of total. Following this Class A + defects were seen in 405 teeth i.e. 29% (231 maxillary and 174 mandibular). Subsequently class B+ defects were seen in 322 teeth i.e. 23% (193 maxillary and 129 mandibular) and finally the class B- defects were seen in 174 teeth i.e. 10% (maxillary 85 and mandibular 89) ( Table 3). Since, the smile line normally extends till premolars and hence, the treatment of these defects in premolars demands further studies. In addition 130 patients out of 150 were using horizontal scrub technique (86.6%) and 12 patients were using tooth powder and tooth brush (8.6%).

**Table 3:**
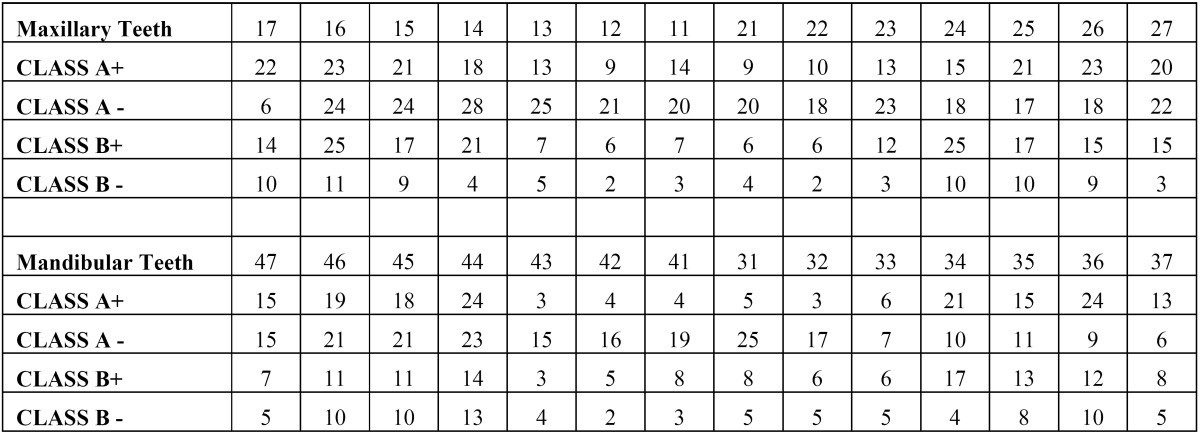
Distribution of root surface defects in maxillary and mandibular arch.

## Discussion

This study aimed at assessing the prevalence of surface defects proposed by Pini Prato *et al.* 2010 in gingival recession areas. This classification was based on identifiable CEJ and presence or absence of cervical defect. In this study, the examination was carried out using a UNC-15 periodontal probe and 2.5 X magnifying loupes which is different from the previous two similar studies in the literature. Besides the sample size is higher in this study compared to the previous two studies where they examined 1000 and 1010 defects respectively (8,12).

A total of 150 patients were examined out of which, males exhibited the highest frequency of these step like defects compared to females, 59% and 41% respectively. This could be attributed to the use of finger and tooth powder or toothpaste as a cleaning aid amongst men (11.9%) when compared to women (4.3%) as suggested by Oberoi *et al.* (13) 2014. Besides, in subjects using tooth-brush as a cleaning aid, males were found to change their brush frequently i.e. once a month than females (13).

The total number of patients examined in this study were lesser compared to Pini Prato study, where they recruited a total of 359 patients (8). Nevertheless the teeth affected were more in this study which is similar to Bhusari P *et al.* (12) 2014. This suggests that in each patient, on an average 9 to 10 teeth with defects were prevalent in Indian population as opposed to western population, where only 2 to 3 teeth were affected per patient. This could be attributed to oral hygiene practices and habits of Indian population. Although this study is conducted in one of the major metro cities of India, the population that we have examined, not necessarily are natives of this major city. They could have migrated at a later age in life from rural areas of India. The use of salt with sea weed, neem stick, charcoal, toothpowder and brick powder using finger is still a prevalent oral hygiene practice in rural areas (14,15).

Heasman PA *et al.* (16) 2015 classified recession into inflammatory and non-inflammatory types. They suggested that tooth brushing is the primary causative factor in the development and progression of non-inflammatory gingival recession.

Thus, this study may not represent only non-inflammatory ‘tooth brush’ trauma type of defect but may reflect other oral hygiene practices causing such defects. 86.6% of the study subjects used horizontal scrub technique to clean their teeth, 8.6% were using toothbrush with tooth powder. Besides it could have been interesting to explore the duration of tooth brush and tooth paste usage and other oral hygiene practices since their childhood, which could have given a greater insight into the causative factor for gingival recession with or without defects (15).

Since, most of the periodontal procedures are performed under magnification in our periodontal clinics, loupes of 2.5 X were easily available to examine these defects. Eichenberger M *et al.* (17) 2011 evaluated the near visual acuity of dentists and concluded that near visual acuity differs among individuals and reduces over a lifetime. Irrespective of age or natural vision, visual acuity can be significantly enhanced by the use of magnifying loupes (18). The 2.5X magnification serves quite well in identifying these defects along with the CEJ. Besides, it has been suggested, enhanced visual acuity has a positive influence on the precision of diagnosis. However, there are no studies to compare different types of magnification devices affecting the diagnostic outcome in dental settings. The investigation of comparing different magnifying devices should be warranted in future studies. For instance, comparing magnifying loupes with magnifying lens as suggested in the previous two studies. The difference in the results of our study compared to the previous studies could have been attributed to the difference in the usage of magnifying devices.

This study suggested that the highest number of defects belonging to the category of A-, i.e. an identifiable CEJ without defect. Although, this study shared a similarity with that of Pini Prato (8) 2010 in the category of A- being the highest among all categories; it was still 10% lower than that of Pini Prato’s. Furthermore, this was dissimilar when compared to the study performed by Bhusari P *et al.* (12) 2014 where they suggested the highest defect category being A+. The class A - category allows a predictable outcome when we consider this classification along with Miller’s Class I and II. Thus, we speculate Miller’s Class I and II with that of Pini Prato’s A- classification should give us 100 % root coverage. Following A-, about 29% belonged to A+ category which was not in agreement with either of the two previously published studies. Collective percentage of B +, B- poses the challenge of placing the flap at the precise CEJ location. Hence, assessment of the treatment outcome is difficult in category B, although the recession is classified under Miller’s class I & II.

Even though, several treatment options are available for these lesions, comparative studies are not available in the literature. These shortcomings make meta-analysis hard and prevent us to reach any evidence-based conclusion about the treatment of choice for these defects. Santamaria MP *et al.* (19) 2013 conducted a study to evaluate the microbiological and immunological influence of subgingival resin modified glass ionomer restoration with or without connective tissue graft (CTG) for the treatment of gingival recession associated with non-carious cervical lesion. They concluded that the presence of this periodontal restorative interphase in the subgingival area may not have a negative impact on subgingival microflora and immunological markers. However, they recommended further trials to test with larger samples and to evaluate with different materials and also associated immunological markers.

Several clinical studies have been cited in the literature with regard to the repair of vertical fractured roots with 4-META/MMA-based adhesive resin. This material exhibited favourable bonding capacity in a wet environment and biocompatibility with cells such as mesenchymal precursors and osteoblasts. Additionally, Satoshi *et al.* (20) 2014 proposed that adhesive materials with the property of delivering growth factors should be investigated in the future to promote tissue regeneration. Similarly, we recommend routine use of restorative materials with the property of promoting tissue regeneration and restoring the defect would become valuable in treating areas of gingival recession with step-like defects (A+ and B+).

Among the surgical modalities to treat the root exposures, coronally advanced flap (CAF) with CTG is considered to offer the best results. However, the major shortcoming of this procedure is morbidity at the donor site. Hence, tissue-engineered materials must be investigated against palatal tissue harvesting (21). Although, CAF with enamel matrix derivative (EMD) (22) has shown to offer better results, the affordability of the material is one of the disadvantages. Thus, CAF with CTG may still continue to remain a gold standard (23).

Pini Prato *et al.* (24) 2004, suggested two different techniques to manage gingival recessions with defects, based on the depth of the step. CAF with CTG for Steps <1mm depth and 2 CTG s for steps >1mm were suggested. The first CTG was exactly to fill the dimensions of the defect without extending laterally and the second CTG over the first one, extending laterally to reach the adjacent connective tissue. Further, this was covered by a CAF. However, randomized clinical trials with a longer follow-up with the periodic assessment of pulpal vitality and patency of the canal needs to be assessed.

Zucchelli G *et al.* (25) 2011, suggested a decision making process for treating the root defects associated with gingival recessions based on the topographic relationship between the maximum root coverage and the step defects. They concluded, CAF, bilaminar procedure, odontoplasty, resoration along with CAF, restoration and CAF alone, and restoration only provided good esthetics and emergence profile in these areas.

This classification is relatively new and as of now very few papers have acknowledged this as a useful and practical classification. However, the management of these surface defects associated with gingival recession would benefit in future in terms of identifying right treatment modalities for each of these defects. Thus, randomized controlled trials to test the effective approach to treat these defects with either restorative techniques or surgical procedures or a combination of the two would be the future direction.

## Conclusions

Within the limits of the study, the following conclusions are made.

• Root surface defects with gingival recession are more prevalent in our population compared to the western population

• Majority of defects were found in maxillary incisors, canines and premolar regions which are in the smile line.

• Horizontal scrub technique was the most prevalent method practiced.
